# Interacting binding insights and conformational consequences of the differential activity of cannabidiol with two endocannabinoid-activated G-protein-coupled receptors

**DOI:** 10.3389/fphar.2022.945935

**Published:** 2022-08-09

**Authors:** Eliud Morales Dávila, Felipe Patricio, Mariana Rebolledo-Bustillo, David Garcia-Gomez, Juan Carlos Garcia Hernandez, Brenda L. Sanchez-Gaytan, Ilhuicamina Daniel Limón, Jose Manuel Perez-Aguilar

**Affiliations:** ^1^ School of Chemical Sciences, Meritorious Autonomous University of Puebla (BUAP), University City, Puebla, Mexico; ^2^ Neuropharmacology Laboratory, School of Chemical Sciences, Meritorious Autonomous University of Puebla (BUAP), University City, Puebla, Mexico; ^3^ Chemistry Center, Science Institute, Meritorious Autonomous University of Puebla (BUAP), University City, Puebla, Mexico

**Keywords:** cannabidiol, G-protein-coupled receptors, all-atom molecular dynamic simulations, G-protein-coupled receptor 55, cannabinoid type-1 receptor

## Abstract

Cannabidiol (CBD), the major non-psychoactive phytocannabinoid present in the plant *Cannabis sativa,* has displayed beneficial pharmacological effects in the treatment of several neurological disorders including, epilepsy, Parkinson’s disease, and Alzheimer’s disease. In particular, CBD is able to modulate different receptors in the endocannabinoid system, some of which belong to the family of G-protein-coupled receptors (GPCRs). Notably, while CBD is able to antagonize some GPCRs in the endocannabinoid system, it also seems to activate others. The details of this dual contrasting functional feature of CBD, that is, displaying antagonistic and (possible) agonistic ligand properties in related receptors, remain unknown. Here, using computational methods, we investigate the interacting determinants of CBD in two closely related endocannabinoid-activated GPCRs, the G-protein-coupled receptor 55 (GPR55) and the cannabinoid type 1 receptor (CB_1_). While in the former, CBD has been demonstrated to function as an antagonist, the way by which CBD modulates the CB_1_ receptor remains unclear. Namely, CBD has been suggested to directly trigger receptor’s activation, stabilize CB_1_ inactive conformations or function as an allosteric modulator. From microsecond-length unbiased molecular dynamics simulations, we found that the presence of the CBD ligand in the GPR55 receptor elicit conformational changes associated with antagonist-bound GPCRs. In contrast, when the GPR55 receptor is simulated in complex with the selective agonist ML186, agonist-like conformations are sampled. These results are in agreement with the proposed modulatory function of each ligand, showing that the computational techniques utilized to characterize the GPR55 complexes correctly differentiate the agonist-bound and antagonist-bound systems. Prompted by these results, we investigated the role of the CBD compound on the CB_1_ receptor using similar computational approaches. The all-atom MD simulations reveal that CBD induces conformational changes linked with agonist-bound GPCRs. To contextualize the results we looked into the CB_1_ receptor in complex with a well-established antagonist. In contrast to the CBD/CB_1_ complex, when the CB_1_ receptor is simulated in complex with the ligand antagonist AM251, inactive conformations are explored, showing that the computational techniques utilized to characterize the CB_1_ complexes correctly differentiate the agonist-bound and antagonist-bound systems. In addition, our results suggest a previously unknown sodium-binding site located in the extracellular domain of the CB_1_ receptor. From our detailed characterization, we found particular interacting loci in the binding sites of the GPR55 and the CB_1_ receptors that seem to be responsible for the differential functional features of CBD. Our work will pave the way for understanding the CBD pharmacology at a molecular level and aid in harnessing its potential therapeutic use.

## 1 Introduction

The endocannabinoid system is a signaling apparatus that regulates a variety of widespread functions in the central nervous system including, synaptic plasticity, learning, sleep, eating, inflammation, immune responses, and pain and temperature control, to mention a few (Lu and MacKie, 2016). The plethora of cellular functions associated with the endocannabinoid systems makes it an attractive pharmacological target. The endocannabinoid system comprises several cellular entities including the endocannabinoids, which are endogenous cannabinoid compounds that modulate the signaling system, as well as various biomacromolecules, i.e., receptors and enzymes. In addition to the enzymes that synthesize and decompose the endocannabinoids, various receptors constitute the endocannabinoid system including two G-protein-coupled receptors, namely, the cannabinoid type 1 (CB_1_) and type 2 (CB_2_) receptors. In addition to CB_1_ and CB_2_, other receptors have been proposed to participate as mediators of the effects of the cannabinoid ligands including the G-protein-coupled receptor 55 (GPR55) (Ryberg et al., 2007; Kapur et al., 2009).

Among the different ligands able to modulate the cannabinoid receptors, those present in the *C. sativa* plant exhibit a significant interest not only because of their role as possible therapeutic agents but also because of their increasing recreational use which may also pose a public health issue (Hall and Lynskey, 2016; Wilkinson et al., 2016). Knowing the details of the stimulation of different cannabinoid receptors by phytocannabinoid compounds may provide pharmacological tools to finely tune the endocannabinoid system. Among the compounds able to modulate the cannabinoid receptors, cannabidiol (CBD), one of the major constituents in the *Cannabis* plant, has attracted significant industrial and medical attention because of its non-psychoactive properties. Yet, the molecular determinants of the CBD interaction with receptors stimulated by endocannabinoids remain poorly understood. In this work and using computational methods, we investigate the interaction of CBD with two endocannabinoid-activated receptors, the GPR55 receptor and the prototypical CB_1_ receptor. While in the former, CBD is known to antagonize the receptor, that is, maintain the receptor’s inactive conformations, the way by which CBD modulates the latter system remains debatable. That is, CBD has been suggested to directly trigger the activation the CB_1_ receptor (Navarro et al., 2020), inactivate the receptor (Grotenhermen, 2004; Batalla et al., 2021; Śmiarowska et al., 2022), or play a role as an allosteric modulator (Shao et al., 2019; Batalla et al., 2021; Austrich-Olivares et al., 2022). First, we looked into the modulation of the CBD ligand on the GPR55 receptor using computational methods. To put our findings in context, we also investigated the GPR55 receptor in complex with a selective agonist. The computational methods utilized are able to capture the ligand-dependent conformational details in the two GPR55 complexes, showing that CBD stabilizes inactive conformations of the receptor while the ML186 agonist favors active-like conformations. Next, we investigated the role of CBD in directly modulating the CB_1_ receptor by placing the ligand in the orthosteric binding site based on the chemical homology of CBD to other CB_1_ ligands (Hua et al., 2017). Our results suggest that CBD is able to stabilize the receptor’s structural changes associated with agonist-bound conformations. Based on these results and similarly to the case of the GPR55, we looked into the CB_1_ receptor in complex with a well-established CB_1_ antagonist, the AM251 ligand. Furthermore, the computational methods utilized here are able to capture the ligand-dependent conformational details in the two endocannabinoid-activated GPCRs and provide a view of the main differential interactions involved in the CBD protein complexes.

## 2 Materials and methods

### 2.1 Homology modeling of the GPCR structures

We decided to model the receptor’s structures of *Rattus norvegicus* to better correlate our findings with the experimental results from our animal models. Additionally, the sequence similarity with *Homo sapiens* is high (around 97% sequence identity in the CB_1_ system and around 74% sequence identity in the GPR55). The sequence for both receptors are shown in Supplementary Figure S1.

### 2.2 GPR55 receptor structures

Thus far, there is no experimentally-determined structural information regarding the GPR55 receptor. Hence, the structure of the rat GPR55 receptor was built using the homology modeling technique (Eswar et al., 2006). To identify possible adequate templates for GPR55, the Swiss-Model server was used (Waterhouse et al., 2018). The selected system was the lysophosphatidic acid receptor 6 (LPA6) which displays a sequence identity with GPR55 of about 30% (PDB ID code 5XSZ). The sequences of the LPA6 template and the rat GPR55 receptors were obtained from UNIPROT (with the codes Q08BG4 and F1MAK4, respectively). The sequences were aligned using BLASTp (Altschul et al., 1990) with subsequent manual adjustments. The pairwise sequence alignment is presented in Supplementary Figure S2. To generate 3D structural models of the rat GPR55 receptor, we used the Modeller 9.23 protocol (Šali and Blundell, 1993). 100 models of the *R. norvegicus* GPR55 receptor were generated and a representative structure was selected based on energetic considerations, that is, using the Modeller’s scoring function (see Supplementary Figure S3).

### 2.3 CB_1_ receptor structures

To model the structures for the rat CB_1_ receptor we used different templates for each receptor’s functional state. In the case of the CB_1_ receptor that will form a complex with CBD (an tentative agonist for CB_1_), the template utilized was the agonist-bound human CB_1_ receptor (PDB ID code 5XRA). In the case of the CB_1_ receptor that forms a complex with AM251 (an antagonist for CB_1_), two structures of the antagonist-bound human CB_1_ receptor were used as templates (PDB ID code 5U09 and code 5TGZ). In both cases, 100 models of the *R. norvegicus* CB_1_ structures were modeled *via* homology modeling using the Modeller 9.23 protocol (Šali and Blundell, 1993). The sequences of the human and rat CB_1_ receptors were obtained from UNIPROT (with the codes P21554 and P20272, respectively). Based on energetic considerations, a representative structure of the rat CB_1_ receptor was selected in each case for further analysis, see Supplementary Figure S4.

### 2.4 CBD/GPR55 complex structures (inactive-like GPR55 system)

To identify possible molecular poses for the CBD/GPR55 complex, blind docking calculations were performed using the Autodock-Vina software (Trott and Olson, 2009). The selected molecular pose for the CBD/GPR55 complex is shown in Supplementary Figure S3. Chemical structure of the ligands are displayed in Supplementary Figure S5.

### 2.5 ML186/GPR55 complex structures (active-like GPR55 system)

Similar to the aforementioned case of the CBD/GPR55 complex, molecular poses for the ML186/GPR55 complex were identified via docking calculations using Autodock Vina (Trott and Olson, 2009). The selected molecular pose for the ML186/GPR55 complex is shown in Supplementary Figure S3.

### 2.6 CBD/CB_1_ complex structures (active-like CB_1_ system)

To predict possible molecular poses for the CBD ligand in the structural framework of the CB_1_ receptor, docking studies were carried out using Autodock Vina (Trott and Olson, 2009). In addition to the energetic considerations (the scoring function of Autodock Vina), we used the structure of the AM11542/CB_1_ complex (PDB ID code 5XRA), as a guide to select a representative structure of the CBD/CB_1_ complex (see Supplementary Figure S4). The structure of the AM11542/CB_1_ complex was selected based on the similarity of the CBD ligand and the AM11542.

### 2.7 AM251/CB_1_ complex structures (inactive CB_1_ system)

Similar docking calculations were utilized in the case of the AM251 antagonist. In this case, in addition to Autodock Vina’s scoring function, the structure of the AM6538/CB_1_ complex (PDB ID code 5TGZ), was used as a guide due to the similarity between the AM251 ligand and the AM6538 ligand. The selected structure is indicated in Supplementary Figure S4.

### 2.8 All-atom molecular dynamics simulations

To provide the interacting ligand binding determinants of the protein systems (CBD/GPR55, ML186/GPR55, CBD/CB_1_, and AM251/CB_1_) and the concomitant conformational consequences, we performed all-atom molecular dynamics (MD) simulations in each of the four protein complexes. Similar general preparation for the systems has been previously used for GPCRs (Perez-Aguilar et al., 2014; Perez-Aguilar et al., 2019; Dror et al., 2011; Lee and Lyman, 2012; Méndez-Luna et al., 2015) and other membrane proteins (Rangel-Galván et al., 2021). Briefly, using the structural information from the docking calculations as the initial complex structure, hydrogen atoms were included representing the most probable protonation states for all the amino acids residues at neutral pH. The protein complexes were embedded in a hydrated symmetric POPC (1-palmitoyl-2-oleoyl-sn-glycero-3-phosphocholine) membrane, where sodium and chlorine ions were included so as to have a physiological salt concentration (0.15 M). Preparation steps that include the imposition of structural force restraints to avoid unrealistic conformation of the system (e.g., water penetration to the lipid membrane) were carried out (Perez-Aguilar et al., 2014; Perez-Aguilar et al., 2019; Rangel-Galván et al., 2021). Next, unbiased MD simulations were carried out for one microsecond (1.0 μs) at a constant temperature and pressure (37°C and 1 atm, respectively).

### 2.9 Class A GPCR position numbering

To define the position of each residue, we will be using the Ballesteros-Weinstein numbering as superscript. In this nomenclature, the first number indicates the transmembrane helix (from one to seven) whereas the second number indicates the residue position relative to the most conserved residue, which is assigned to the number 50 (Ballesteros and Weinstein, 1995).

### 2.10 Structural analysis

The analyses herein were performed using the program VMD and in-house TCL scripts (Humphrey et al., 1996).

## 3 Results

To shed light on the differential activity of CBD in the two endocannabinoid-activated receptors aforementioned, we utilized computational techniques to characterize four protein complexes, CBD/CB_1_, ML186/GPR55, CBD/GPR55, and AM251/CB_1_.

### 3.1 Interacting determinants in the CBD/GPR55 and ML186/GPR55 complexes

#### 3.1.1 The CBD/GPR55 system

**FIGURE 1:**
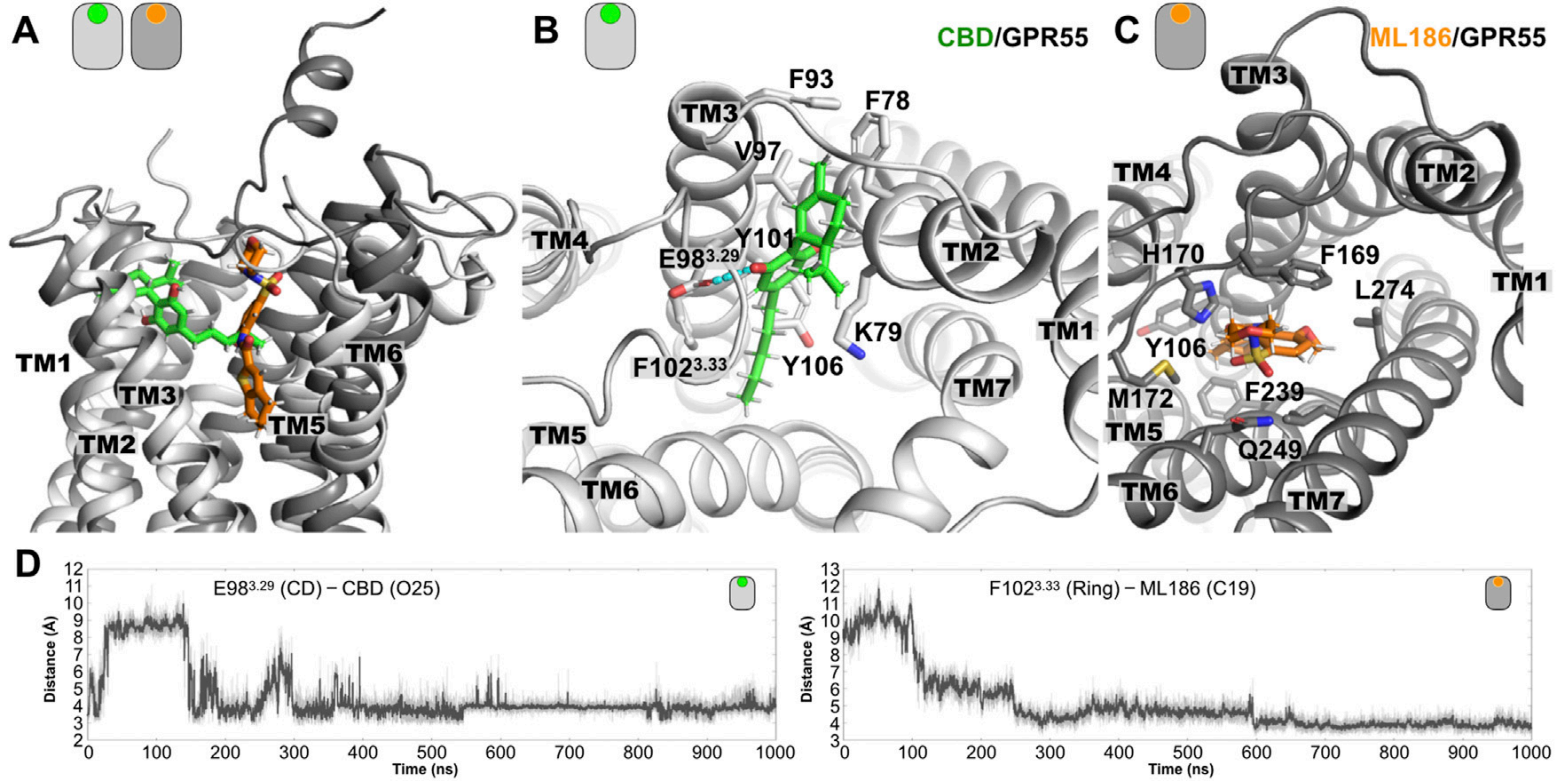
Representative poses from the MD simulations of the GPR55 complexes. **(A)** Lateral view of superimposed structures of the CBD/GPR55 and ML186/GPR55 complexes. Relative to the position of the CBD, the position of the ML186 selective ligand is placed deeper into the TM region of the ligand binding site. **(B)** Extracellular view of the CBD/GPR55 complex, where we can see that the two-ring moiety of CBD inserts in between TM2 and TM3. Flanking this part of the ligand, we observed two charged residues, K79^2.60^ and E98^3.29^. While the former interacts via the aliphatic part of its side chain, the latter forms interactions with one of the CBD hydroxyl (OH) groups (see dashed line). Residues forming the CBD binding site in GPR55 are indicated. **(C)** Extracellular view of the ML186/GPR55 complex. Contrary to the location of CBD in the GPR55 binding site, the selective agonist mainly contacts residues located TM3, TM6, and TM7. Three aromatic residues are part of the residues forming the binding site of ML186. F102^3.33^ places along the main axis of the ligands where it established aromatic interactions with the highly delocalized electron (aromatic) region of the ligand. As for residues F239^6.48^ and Y106^3.37^, they formed the lower part of the agonist binding site. Note that position 6.48 has been denominated toggle switch and has been implicated in class A GPCR activation. Lastly, the SO group of the ligands established polar interactions around residue Q249^6.58^. **(D)** Time evolution plots of the distance between acidic residue E98^3.29^ with one of the CBD hydroxyl (OH) groups as well as between the center of the aromatic sidechain of F102^3.33^ and the C19 atom.

As described in the Methods section, the initial model of the CBD/GPR55 was obtained from docking approaches using the crystal structure of the lysophosphatidic acid receptor 6 (LPA6, 5XSZ.pdb) to model the structure of the GPR55 receptor. The initial structure of the model is shown in Supplementary Figure S3 and was used as the starting point to investigate the CBD/GPR55 complex by unbiased all-atom MD simulations. Figures 1A,B shows a representative final complex structure after 1 μs simulation, where the interactions that stabilized the CBD molecular pose are displayed.

The molecular pose of CBD in the ligand binding site of GPR55 is located mainly in the vicinity of TM2 and TM3, and close to the conserved disulfide bond formed by C94^3.25^ and C168^ECL2^. In particular, the terpenoid ring moiety of CBD inserts in between TM2 and TM3 as indicated in Figure 1B. In this position, the CBD ligand is flanked by two charged residues, i.e., K79^2.60^ and E98^3.29^. CBD forms polar interaction with the side chain of E98^3.29^ *via* one of the hydroxyl (OH) groups. The time evolution of this distance along the entire MD simulations is shown in Figure 1D. As for residue K79^2.60^, it interacts with CBD using its aliphatic side chain [(CH_2_)_4_]. Residues that delineate the ligand binding site of CBD are indicated in Figure 1B.

#### 3.1.2 The ML186/GPR55 system

To put our findings in context, we also investigate the interactions of the GPR55 selective agonist ML186. A representative structure of the ML186/GPR55 complex at the final stages of the 1 μs simulation is illustrated in Figures 1A,C. In contrast to the location of the CBD ligand in the GPR55 binding site, the ML186 agonist positions in proximity to TM3, TM6 and TM7. The binding site of the selective agonist is partially formed by three bulky aromatic residues, namely F102^3.33^, Y106^3.37^, and F239^6.48^. F102^3.33^ positions along the main axis of the ligand where it established aromatic interactions with the highly delocalized electron (aromatic) region of the molecule, see the right panel in Figure 1D. Residues Y106^3.37^ and F239^6.48^ formed the lowest region of the ligand binding site. In particular, position 6.48 has been denominated “toggle switch” and associated with class A GPCR activation (see below for more details). The sulfonyl functional group (SO_2_) group of the agonist forms polar interactions with residue Q249^6.58^ in the extracellular part of TM6. Finally, in comparison with the relatively shallow position of the CBD, the ML186 agonist places into a deep molecular pose in the GPR55 ligand binding site (Figure 1A).

### 3.2 Structural consequences observed in the GPR55 receptor by the presence of the ligands

**FIGURE 2:**
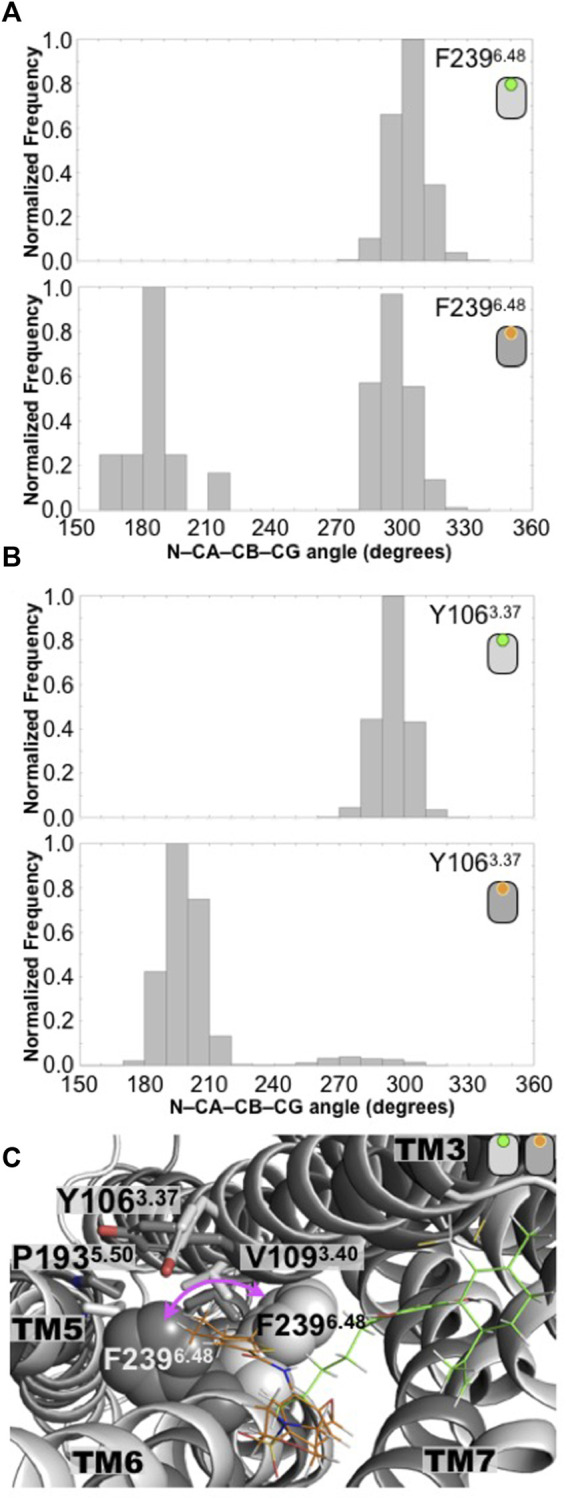
Conformation changes in the toggle switch motif distinguished the antagonist-bound versus the agonist-bound GPR55 complexes. **(A)** Distributions of the N-CA-CB-CD dihedral angle of the F239^6.48^ residue in the CBD/GPR55 and ML186/GPR55 complexes. Position 6.48 is known as the toggle switch and has been implicated in GPCR activation. In the case of the GPR55, position 6.48 bears a phenylalanine residue, F239^6.48^. **(B)** Another residue that seems to play a role in the changes of F239^6.48^ rotameric state is the Y106^3.37^, which also displays a distinctive dihedral angle distribution. **(C)** Superimposed final structures of the CBD/GPR55 and ML186/GPR55 simulations. The different position of residue F239^6.48^ in the two GPR55 systems is illustrated (magenta arrow). The deeper molecular pose of the selective agonist ML186 (orange) may be responsible for the different position of the F239^6.48^ sidechain.

In order to understand the structural consequence of the presence of CBD ligand in the receptor’s conformations, we analyze several structural motifs associated with GPCR activation. One particular locus linked with GPCR activation is position 6.48, the so-called toggle switch, which usually bears the aromatic residue tryptophan (W^6.48^). Upon activation, there is a structural rearrangement in the vicinity of this residue, that seems to propagate conformational changes towards the intracellular side (Zhou et al., 2019). At this position, the GPR55 receptor contains another aromatic residue, the F239^6.48^. In this context, we analyzed the conformations of this residue in the CBD-bound and ML186-bound GPR55 systems, particularly the rotameric states explored by the bulky F239^6.48^ residue. As observed by the dihedral angles values of the angle defined by the N-CA-CB-CG atoms, in the case of the CBD/GPR55 complex, the distribution of the values explored by the receptor along the simulated trajectory are mainly distributed around 280°–320° (unimodal distribution, see Figure 2A). In the case of the agonist-bound GPR55 complex, the presence of the ML186 ligand provokes a change in the position of the F239^6.48^ residue, as indicated by the bimodal distribution of the N-CA-CB-CG dihedral angle (Figure 2A). Experimental evidence indicates that a more dynamic and heterogeneous conformational behavior of the receptor is attributed to the binding of an agonist ligand (Manglik et al., 2015; Latorraca et al., 2017). It this case, the bimodal distribution observed in the ML186/GPR55 system at this position may be associated with active-like conformations while the unimodal distribution of this dihedral angles in the CBD/GPR55 complex are attributed to a GPCR’s inactive conformations. This behavior may be attributed to the thiophene-containing fused-rings moiety of the ML186 agonist, which directly contacts the side chain of the F239^6.48^ residue (Figure 1A). These results are in good agreement with the current notion that CBD is an antagonist of the GPR55 receptor while ML186 exhibits agonistic function on the GPR55 (Ryberg et al., 2007; Ross, 2009; Sharir and Abood, 2010).

Additionally, it has been suggested that conformational changes in position 6.48 with residues in TM3 and TM6 occur upon activation (Zhou et al., 2019; Hilger, 2021). Our simulations suggest that the conformational changes of the F239^6.48^ residue are in close relation with those of two other aromatic residues, the bulky aromatic residues Y106^3.37^ and F235^6.44^. Analysis of the equivalent Y106^3.37^ dihedral angle (N-CA-CB-CG atoms) indicates a drastic change in the rotameric states sampled by this residue. While in the CBD/GPR55 system, which displays the unimodal distribution of F239^6.48^, the Y106^3.37^ dihedral angle (N-CA-CB-CG atoms) also indicates that this aromatic residue samples a one-peak distribution conformations (Figure 2A). The more dynamic behavior of F239^6.48^ causes a significant change in the distribution of rotameric states that explores the Y106^3.37^ dresidue (Figure 2B).

Lastly, the changes in the GPR55 toggle switch causes F235^6.44^ to follow similar trend, that is, the CBD/GPR55 displays an unimodal distribution of the dihedral angle define by the N-CA-CB-CG atoms while the ML186/GPR55 complex displays a more heterogenic distribution (Supplementary Figure S6).

The significant conformational response of the GPR55 receptor to the presence of the CBD antagonist and the ML186 agonist on structural motifs known to play a role on activation, seems to be in good agreement with the experimental evidence linked to these two ligands [Ryberg et al., 2007; Ross, 2009; Sharir and Abood, 2010; Kotsikorou et al., 2013; Anderson et al., 2021; Heynen-Genel et al., 2010 September 29 (updated 26 May 2011); Sharir. et al., 2012; Kotsikorou. et al., 2011]. Furthermore and regarding the known modulatory properties of the two ligands, CBD (antagonist) and ML186 (agonist), our computational approaches were able to distinguish the distinct GPR55 conformations elicited by the presence of each ligand (Figure 2C).

**FIGURE 3:**
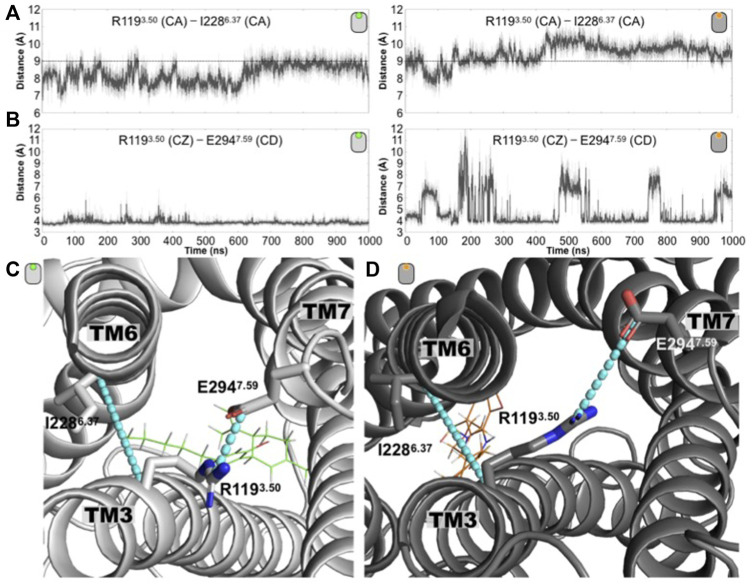
Conformation changes around the conserved intracellular residue R3.50 distinguished the antagonist-bound versus the agonist-bound GPR55 complexes. **(A)** Time evolution of the R119^3.50^ (CA)—I228^6.37^ (CA) distance for the antagonist-bound (left) and agonist-bound (right) GPR55 systems. The dashed line at 9 Å is drawn to guide the eye. **(B)** Time evolution of the distance R119^3.50^ (CZ)—E294^7.59^ (CD) distance for the antagonist-bound (left) and agonist-bound (right) GPR55 complexes. This electrostatic interaction remains in the former while in the latter is frequently disrupted. **(C)** Representative structures of the **(C)** CBD/GPR55 and the **(D)** ML186/GPR55 complexes are shown where the two plotted distances are illustrated as cyan lines.

In addition to the aforementioned toggle switch residue, another structural feature that is a hallmark of class A GPCR activation is the presence of an charge-charge intracellular interaction, the so-called ionic lock, formed by R^3.50^ and D/E^6.30^ (Zhou et al., 2019). Upon activation, this ionic lock needs to be broken so that an outward shift of TM6 is possible (Zhou et al., 2019). Although there is an arginine residue at position 3.50 in GPR55 (R119^3.50^), no acidic residue is present at position 6.30. Nevertheless, to have a sense of the relative displacement of the intracellular segment of TM6 from TM3, we calculate the distance between the CA atoms of R119^3.50^ and I228^6.37^. Position 6.37 was selected since its CA atom is located around the same plane as the CA atom from position 3.50. As indicated in Figure 3A, the R119^3.50^ (CA) and I228^6.37^ (CA) distance in the antagonist-bound receptor barely samples values longer than 9 Å. In contrast, in the agonist-bound receptor, most of the time this distance overpasses the 9 Å value even reaching 11 Å values. Our calculations reveal that R119^3.50^ forms a salt bridge interaction with residue E294^7.59^. Indicative of this interaction, the time evolution of the R119^3.50^ (CZ)—E294^7.59^ (CD) distance is shown in Figure 3B. In the CBD/GPR55 complex, the salt-bridge interaction remains very stable along the entire simulation, however, in the case of the ML186/GPR55 system, the break of this interaction is frequently observed. Again, the computational approaches capture distinctive conformations adopted by the receptor in the presence of different ligands, either antagonist or agonist (Figures 3C,D).

Hence, in the case of the GPR55, the structural consequences of the presence of CBD suggests that the ligand stabilizes the receptor’s inactive conformations, which is in good agreement with the suggested antagonistic activity of CBD on the GPR55 (Ryberg et al., 2007; Ross, 2009; Sharir and Abood, 2010). On the other hand, the presence of the selective ML186 agonist favors structural changes linked to GPCR’s active-like conformations, again, in good agreement with the functional properties of the ligand [Kotsikorou et al., 2013; Anderson et al., 2021; Heynen-Genel et al., 2010 September 29 (updated 26 May 2011); Sharir et al., 2012; Kotsikorou et al., 2011)

### 3.3 Interacting determinants of the CBD ligand in the CB_1_ receptors

Since our computational approaches were able to distinguish the conformational consequences of each of the ligands, either agonist or inverse agonist, for the case of the GPR55 receptor, we decided to apply similar approaches to shed light on the yet-unclear functional activity of CBD on the prototypical CB_1_ receptor. Based on the structural similarity of CBD with different cannabinoid ligands (Hua et al., 2017) including the structure of the phytocannabinoid delta-9-tetrahydrocannabinol (∆^9^-THC), which exhibits partial agonism at the CB_1_ receptor (Pertwee, 2008; Dutta et al*.*, 2022a), we decided to select molecular poses from our docking calculations that places the CBD compounds in the orthosteric binding site of CB_1_.

**FIGURE 4:**
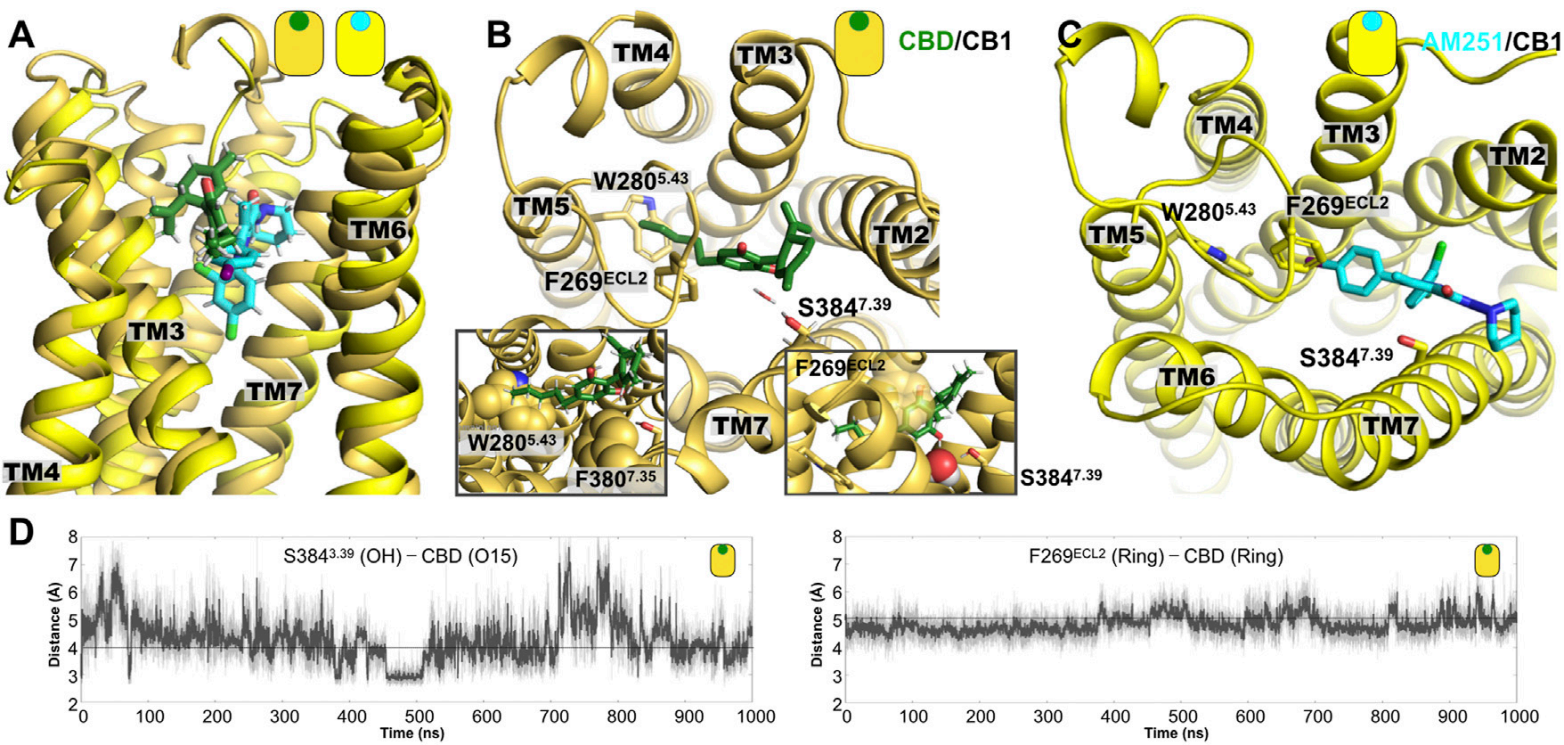
Representative poses from the MD simulations of the CB_1_ complexes. **(A)** Lateral view of superimposed structures of the CBD/CB_1_ and AM251/CB_1_ complexes. **(B)** Extracellular view of the CBD/CB_1_ complex, where we can see the interaction of the aromatic rings of CBD and the side chain of residue F269^ECL2^. A water-mediated polar interaction of one of the CBD hydroxyl (OH) groups is formed with the side chain of residue S384^7.39^. The left inset shows the aromatic interaction with the side chain of residue F269^ECL2^ and with the side chain of F380^7.35^. The right inset shows in more detail the water molecule that mediates the interaction of CBD with the residue S384^7.39^. Mutation of these two residues in TM7 has been shown to reduce agonist potency of cannabinoid-like ligands. **(C)** Extracellular view of the AM231/CB_1_ complex. **(D)** Time evolution plots of the distance between one of the CBD hydroxyl (OH) groups and the hydroxyl group from S384^7.39^ and the ring-ring distance between the aromatic ring of CBD and the side chain of residue F269^ECL2^.

As described in the Methods section, the initial model of the CBD/CB_1_ was obtained from docking approaches. In this case, the information from the crystal structure of the CB_1_ receptor in complex with an agonist ligand that shares structural similarities with the CBD ligand (5XRA.pdb accession code), was used as reference to evaluate the ligands poses (Supplementary Figure S5). The CBD/CB_1_ system was investigated by unbiased all-atom MD simulations using protocols previously described for transmembrane proteins (see Methods). Figure 4A displays a representative final structure of both CBD complexes at the final stages of the 1 μs simulation. Residues that delineate the CBD binding site are also indicated.

In the case of the CBD/CB_1_ complex, the main interactions that stabilized the ligand in the putative orthosteric binding site of CB_1_ are indicated. The time evolution of distance representing two of the main interactions are indicated in Figure 4D. As observed, most of the interactions are aliphatic with a particularly relevant aromatic interactions between the aromatic ring of CBD and the side chain of residue F269^ECL2^, which is located in the extracellular loop 2 (right inset Figure 4B) and the side chain of residue F380^7.35^ in TM7 (left inset Figure 4B). The relevance of the latter interaction has been documented for CB_1_ agonists, showing the mutation for alanine reduces agonist’s potency (Hua et al., 2017). Interestingly, the alkyl tail of CBD directly contacts the bulky side chain of residue W280^5.43^ in TM5. Lastly, one of the OH groups in CBD forms a polar interaction, sometimes mediated by a water molecule, with residue S384^7.39^ (insets Figure 4B). Notably, the presence of this polar residue, S384^7.39^, has been found to be relevant in the interaction of CB_1_ with cannabinoid-like agonists, since the removal of the hydroxyl group (mutation for alanine) reduced the potency of this type of ligands (Hua et al., 2017).

### 3.4 Structural consequence observed by the presence of CBD in the CB_1_ receptor

The presence of CBD would elicit conformational changes in the CB_1_ that stabilize either active-like or inactive conformations (Navarro et al., 2020; Grotenhermen, 2004; Batalla et al., 2021; Śmiarowska et al., 2022; Pertwee, 2008).

**FIGURE 5:**
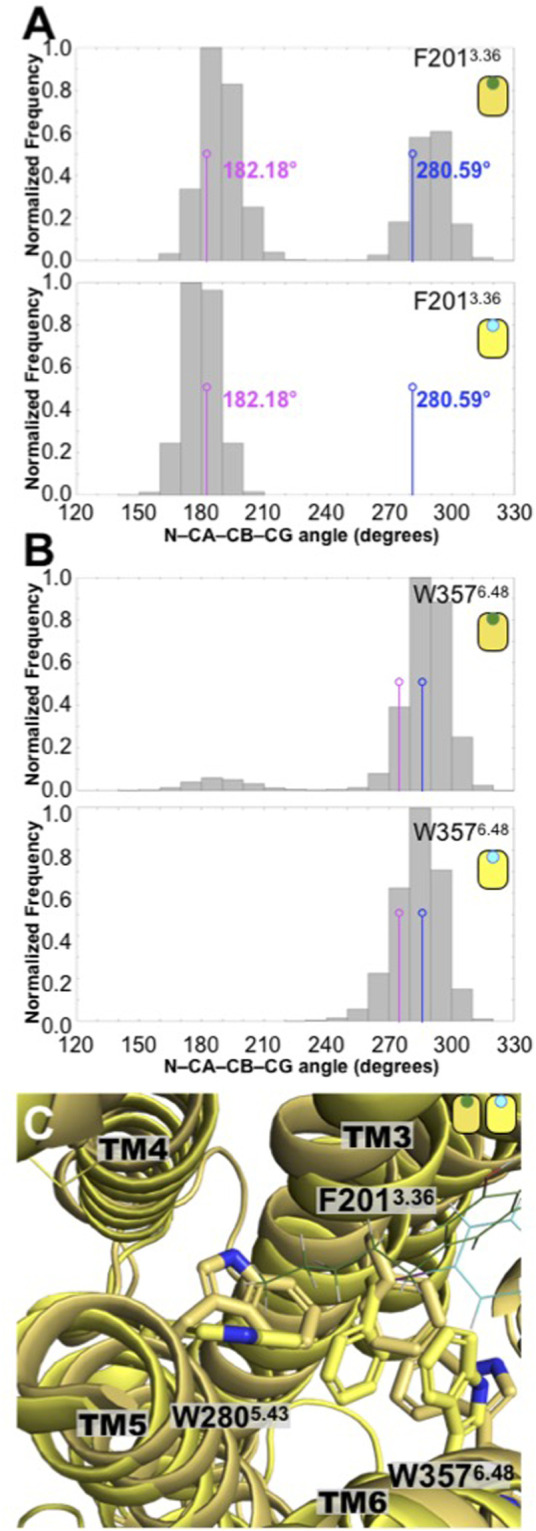
Conformation changes in the toggle switch F201^3.36^ and W357^6.48^ residues displayed different distributions in the CBD/CB_1_ and AM251/CB_1_ complexes. **(A)** Distributions of the N-CA-CB-CD dihedral angle of the F201^3.36^ residue in the CBD/CB_1_ and AM231/CB_1_ complexes. The values indicated in magenta correspond to the antagonist-bound CB_1_ structure (5TGZ.pdb) while those colored in blue correspond to the agonist-bound CB_1_ structure (5XRA.pdb). As seen here, only the CBD complex explored conformations of the twin toggle switch associated with the agonist-bound CB_1_ structure. **(B)** Distributions of the N-CA-CB-CD dihedral angle of the W357^6.48^ residue in the CBD/CB_1_ and AM231/CB_1_ complexes. Together these two residues are known as the twin toggle switch. **(C)** Superimposed final structures of the CBD/CB_1_ and AM251/CB_1_ complexes. The different rotameric states of the F239^3.36^, W357^6.48^, and W280^5.43^ residues in the two CB_1_ complexes are shown. As indicated in the main text and in Figure 6, the alkyl tail of CBD directly contacts the bulky side chain of residue W280^5.43^.

To see the consequences of the presence of CBD on the CB_1_, first we analyzed the conformation of two bulky hydrophobic residues, which has been linked to CB_1_ activation, that is, the F201^3.36^ and W357^6.48^ residues. These two “bulky” residues are sometimes denominated a “twin toggle switch,” since a conformational change in their rotameric state has been proposed to be essential for the receptor activation (Hua et al., 2017). The distribution of the dihedral angle values formed by the (N-CA-CB-CG atoms of each residue are plotted in Figure 5. We can see that, in the presence of CBD, the F201^3.36^ residue explores two distinctive values (bimodal distribution). The first distribution peak overlaps with the value of the antagonist-bound CB_1_ receptor [5TGZ.pdb; F^3.36^(N-CA-CB-CG) dihedral angle of 182.18°] (Hua et al., 2016), however, the second distribution peak overlaps with the value observed in the agonist-bound CB_1_ receptor [5XRA.pdb; F^3.36^(N-CA-CB-CG) dihedral angle of 280.59°] (Hua et al., 2017). To put our results in context, we investigated the CB_1_ receptor in complex with a well-known antagonist, the AM251 ligand using atomistic MD simulations (Figure 4C). In stark contrast with the CBD/CB_1_ case, in the presence of the AM251 antagonist, only one conformation was observed (unimodal distribution) that overlaps with the antagonist-bound CB_1_ receptor. This particular conformation of the F^3.36^ has been associated with the inactive state of the receptor. Moreover, in the presence of CBD, the W357^6.48^ residue also explores two distinct conformations (bimodal distribution) although the first peak is barely sampled by the system. Once again, in the presence of the AM251 antagonist, only one conformation was observed, which has been associated with the inactive state of the receptor (unimodal distribution). Finally, this conformational difference in the residues that constitute the twin toggle switch are related with the rotameric states explored by the bulky residue W280^5.35^. As observed in Figure 4B, the alkyl tail of CBD contacts the side chain of W280^5.35^ which could facilitate the changes identified in the twin toggle switch. Hence, the presence of CBD causes the receptor to adopt active-like conformations (twin toggle switch) that are associated with agonist-bound systems. In contrast, the results for the simulation of the AM251/CB_1_ complex indicate that the conformations adopted by the receptor in the presence of the antagonist ligand are linked to inactive conformations of the receptors. Thus, our computational methods correctly differentiate the structural changes in CB_1_ elicit by the different ligands, that is, CBD and AM251 (CB_1_ antagonist). Moreover, our results suggest that CBD may act as an agonist on the CB_1_ receptor.

**FIGURE 6:**
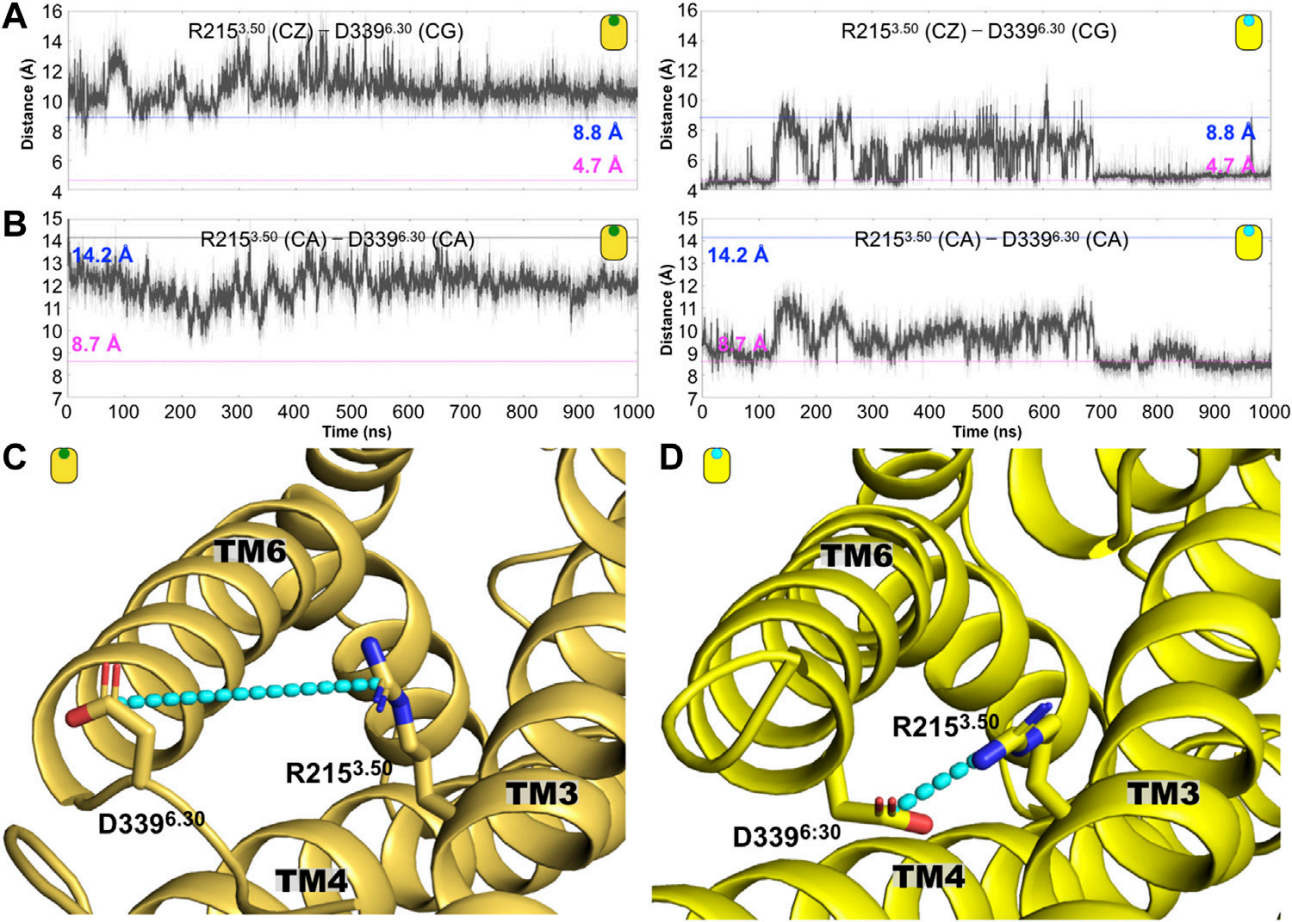
Conformation changes around the conserved intracellular residue R3.50 distinguished the antagonist-bound versus the agonist-bound CB_1_ complexes. **(A)** Time evolution of the R119^3.50^ (CZ)—D339^6.30^ (CG) distance for the CBD-bound (left) and antagonist-bound (right) CB_1_ systems. The dashed line at 4.7 (magenta) and 8.8 Å (blue) indicated corresponding distance in the antagonist-bound (5TGZ.pdb) and agonist-bound (5XRA.pdb) CB_1_ structures, respectively. **(B)** Time evolution of the distance R119^3.50^ (CA)-D339^6.30^ (CA) distance for the CBD-bound (left) and antagonist-bound (right) CB_1_ complexes. This electrostatic interaction is disrupted in the former while in the latter is frequently formed. **(C)** Representative structures of the **(C)** CBD/CB_1_ and the **(D)** AM231/CB_1_ complexes are shown where the ionic lock is illustrated as cyan lines.

As mentioned before, one of the hallmarks of GPCR activation is the polar interaction commonly formed by two charged residues located at the intracellular segment of TM3 and TM6. In the case of the CB_1_ receptor, this so-called ionic lock is formed by residues R215^3.50^ and D339^6.30^ (see figure Figure 6). As indicated in Figure 6A, along the simulation time, we observed the disruption of the ionic lock, which has been established as a prerequisite for the interaction of the GPCR with its respective G-protein. Indicated by the distance between the R215^3.50^(CZ)-D339^6.30^(CG), our results indicate that the values for this distance remains larger that 8.8 A, which is the distance observed in the case of the agonist-bound CB_1_ complex (AM11542/CB_1_, PDB ID code 5XRA). This result indicates that in the presence of CBD, the ionic lock displays conformations associated with agonist-bound states. Similarly, we calculate the same distance, R215^3.50^(CZ)-D339^6.30^(CG), for the case of the AM251/CB_1_ complex (the antagonist-bound system). As indicated in Figure 6A, the ionic lock remains close, around 4.5/4.7 Å (distances observed in antagonist-bound CB_1_ complexes, 5TGZ.pdb/5U09.pdb) and almost never explores distances longer that 8.8 Å. Along the same lines, the R215^3.50^(CA)-D339^6.30^(CA) also indicates the formation of the ionic lock in the antagonist-bound system (AM251/CB_1_ complex) but not in the case of the CBD/CB_1_ complex, again, suggesting that CBD may act as an agonist on the CB_1_ receptor (Figure 6B). Structures illustrating the ionic lock motif for the CBD/CB1 and AM231/CB1 complexes are shown in Figures 6C,D, respectively.

### 3.5 The unbiased MD simulations suggest a sodium-binding site located in the extracellular domain of the CB_1_ receptor

**FIGURE 7:**
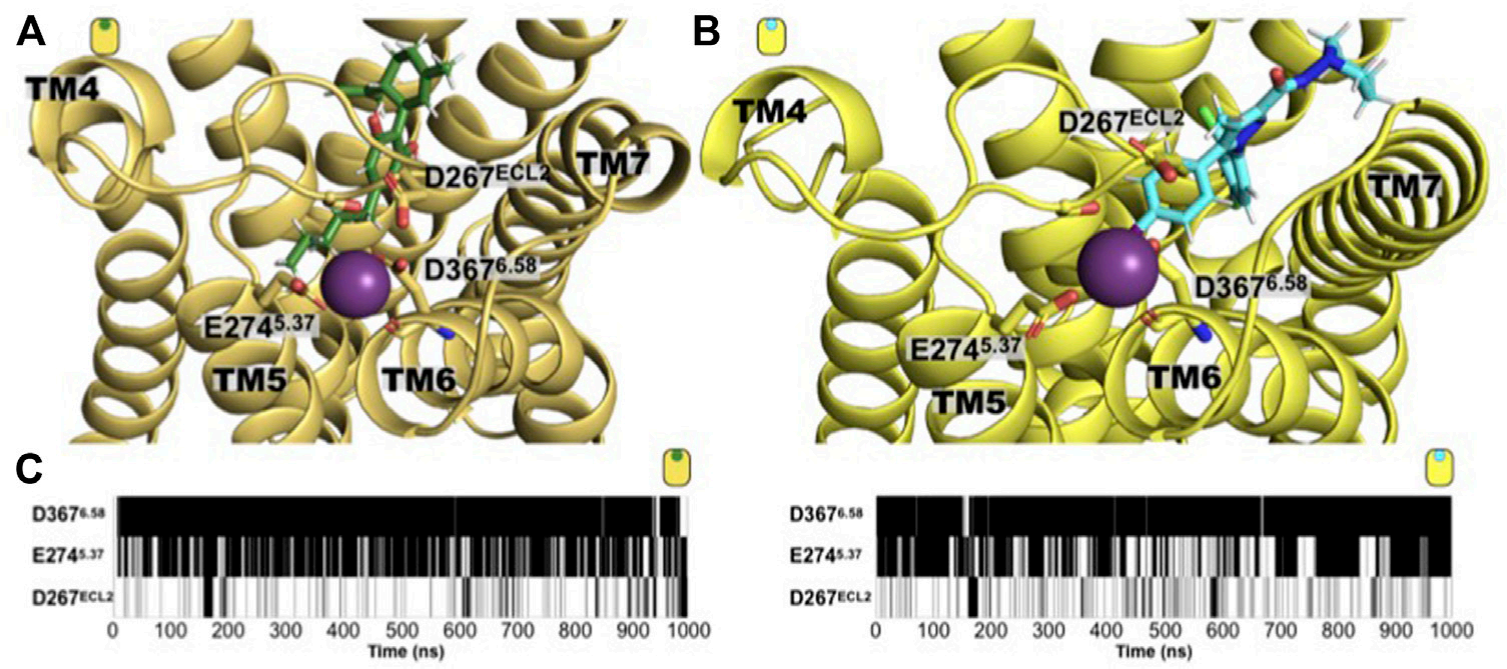
An extracellular sodium-binding site is observed in the simulation of both CB_1_ complexes. Extracellular view of the sodium-binding site identified by our unbiased simulations in the extracellular side of the **(A)** CBD/CB_1_ and **(B)** AM251/CB_1_ receptor. The residues that constitute the binding site are: D367^6.58^, E274^5.37^, and D267^ECL2^. **(C)** Time evolution maps of sodium contact by these three residues where black indicated a contact with a sodium ion (define by a distance of 4 Å or less of any residue heavy atom with a sodium atom), while white indicates the absence of such contact. As shown, residue D367^6.58^ is the main responsible for establishing electrostatic interactions with a sodium cation and to do so, it usually utilizes both, its side chain carboxylate group and its backbone carbonyl group. The acidic nature of the residue bore at these positions is conserved in the human CB_1_ receptor (D367^6.58^, E274^5.37^, and D267^ECL2^ are the positions in rat with the corresponding human positions, D366^6.58^, E273^5.37^, and D266^ECL2^).

Additionally to the detailed description of the ligand binding determinants and the receptor’s structural consequence, our simulations also identified a sodium-binding site located in the extracellular side. Previously, the location of a binding-site at the TM domain of the CB_1_ receptor has been identified (Dutta et al., 2022b; Katritch et al., 2014). From our unbiased MD simulations and in both CB_1_ complexes, we observed the formation of this extracellular sodium-binding site formed by residues located in the extracellular segment of TM5 and TM6, and the extracellular loop 2. D267^ECL2^, E274^5.37^, and D367^6.58^. As shown in Figure 7, along the microsecond MD simulations, the residues D267^ECL2^, E274^5.37^, and D367^6.58^ are in contact with a sodium ion forming a sodium-binding site at the receptor’s extracellular side, regardless of the characteristics of the ligand. The functional consequences of this site need to be further evaluated for a possible exploitation of this tentative allosteric site. Interestingly, no sodium-binding site at the extracellular site was observed in the case of the GPR55 systems.

## 4 Discussion

An integral description of the protein’s function is fundamental to understand the involvement of these biomacromolecules in different cellular activities. Moreover, discerning how different biochemical entities, including exogenous ligands, ions, peptides, and lipids, to mention a few, regulate the protein’s function is needed to finely modulate the cellular response of the proteins.

In this context, computational methods offer tailored tools adapted to investigate at a molecular detail, the function of different biomacromolecules, including proteins. Additionally, computational-based techniques also provide detailed information of the way protein function is modulated by the aforementioned biochemical entities (Almeida et al., 2017; Klepeis et al., 2009; Arinaminpathy et al., 2009; Samish et al., 2011; Hollingsworth and Dror, 2018; Pagadala et al., 2017).

Here we exploit the power of atomistic computer simulations to investigate the role of cannabidiol (CBD), a major cannabinoid compound from the *Cannabis* plant that is closely-related to the well-known delta-9-tetrahydrocannabinol (∆^9^-THC), but lacks its psychoactivity. CBD has attracted great attention in recent years as a possible pharmacological tool to treat different neurological disorders. Additionally, the growing use of CBD as a recreational substance makes it important to understand how this compound interacts with different cellular entities. We applied unbiased all-atom molecular dynamics simulations to investigate the interacting determinants as well as the conformational consequences of CBD in two closely related endocannabinoid-activated GPCRs, the G-protein-coupled receptor 55 (GPR55) and the cannabinoid type 1 receptor (CB_1_). Our results in the GPR55 complexes indicate that the computational methods correctly differentiate the structural changes associated with the presence of each ligand, an antagonist (CBD) and an agonist (ML186). That is, the utilized computational methods identified conformational changes in the GPR55 receptors associated with antagonist-bound receptors (the case of the CBD/GPR55 complex) and agonist-bound receptors (the case of the ML186/GPR55); this is in good agreement with the known modulatory function of these ligands in the GPR55 receptor. Prompted by these results, we subsequently applied similar computational methods to shed light into the role of CBD in the CB_1_ receptor, one of the most abundant GPCRs receptors in the CNS. Our results suggest that the presence of CBD causes conformational changes in the CB_1_ linked to agonist-bound systems. Currently, the role of CBD in the CB_1_ receptor remains debatable, i.e., CBD has been suggested to activate the receptor, to favor CB_1_ inactive conformations or to function as an allosteric modulator. From our investigation we propose that CBD triggers activation of the CB_1_ receptor, nonetheless, we cannot exclude the possibility that CBD may bias either homo- or hetero-dimerization of the CB_1_ receptor and consequently regulate its function in a different way as the agonistic role proposed here. Furthermore, if CBD indirectly modulates the function of the CB_1_, it also remains a possibility that our results cannot be excluded. Yet, we are providing strong evidence that CBD is able to trigger changes in the CB_1_ receptor associated with agonist-bound GPCRs. The detail of our work aids to suggest an unknown sodium-binding site located at the extracellular side of the receptor and formed mainly by residues located at the extracellular termini of TM5 and TM6.

Our investigation shows that computational-based methods are able to correctly distinguish the conformational changes attributed to either an agonist-bound or antagonist-bound GPCRs.

## Data Availability

The datasets presented in this study can be found in online repositories. The names of the repository/repositories and accession number(s) can be found in the article/Supplementary Material.
