# Supplementary material for: Interacting binding insights and conformational consequences of the differential activity of cannabidiol with two endocannabinoid-activated G-protein-coupled receptors
Source: Front Pharmacol. 2022 Aug 9;13:945935. doi: 10.3389/fphar.2022.945935 (PMC9395587; doi:10.3389/fphar.2022.945935)
Supplement: Supplementary file 1 [file DataSheet1.PDF]

Supplementary Material

Interacting binding insights and conformational consequences of the differential activity of cannabidiol with two endocannabinoid-activated G-protein-coupled receptors

Eliud Morales Dávila[1], Felipe Patricio[2], Mariana Rebolledo-Bustillo[1], David Garcia-Gomez[1], Juan Carlos Garcia Hernandez[1], Brenda L. Sanchez-Gaytan[3], Ilhuicamina Daniel Limón[2], Jose Manuel Perez-Aguilar[1,*].

[1] School of Chemical Sciences, Meritorious Autonomous University of Puebla (BUAP), University City, Puebla, 72570, Mexico
[2] Neuropharmacology Laboratory, School of Chemical Sciences, Meritorious Autonomous University of Puebla (BUAP), University City, Puebla, 72570, Mexico
[3] Chemistry Center, Science Institute, Meritorious Autonomous University of Puebla (BUAP), University City, Puebla, 72570, Mexico

jmanuel.perez@correo.buap.mx

1.1 Supplementary Figures

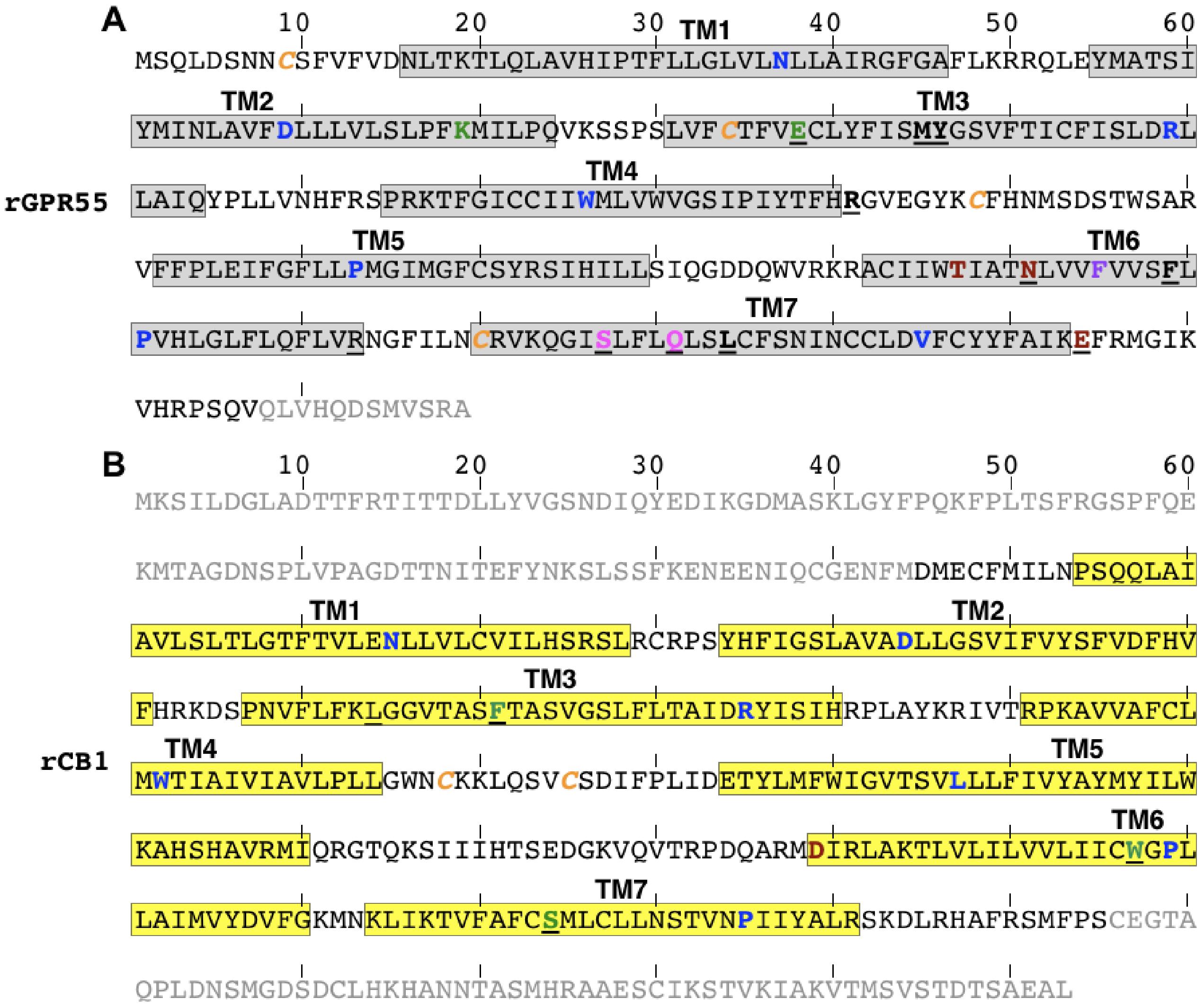

Supplementary Figure 1. Amino acid sequence of the CB1 and GPR55 studied in this work. (A) Aminoacid sequence of the G-protein-coupled receptor 55, GPR55, from *Rattus norvegicus* (UNIPROT ID F1MAK4). Segments excluded from the GPR55 structural model are colored in gray. The transmembrane helices are indicated by gray rectangles while the most conserved residue in each TM is colored blue. Cysteine residues forming a disulfide bond are indicated in orange. (B) Aminoacid sequence of the cannabinoid type 1 receptor, CB1, from *Rattus norvegicus* (UNIPROT ID P20272). The segments excluded from the CB1 model are colored in gray. The transmembrane helices are indicated by yellow rectangles and the most conserved residue in each TM is colored blue.

```
                                                 TM1
LPA6    --------------DNFKYPLYSMVFSIVFMVGLITNVAAMYIFMCSLKLR----NETTT
GPR55   MSQLDSNNCSFVFVDNLTKTLQLAVHIPTFLLGLVLNLLAIRGFGAFLKRRQLEYMATSI
                    TM2                                     TM3
LPA6    YMMNLVVSDLLFVLTLPLRVFYFVQQNWPFGSLLCKLSVSLFYTNMYGSILFLTCISVDR
GPR55   YMINLAVFDLLLVLSLPFKMILPQVKSSP-SLVFCTFVECLYFISMYGSVFTICFISLDR
                                      TM4
LPA6    FLAIVYPFRSRGLRTKRNAKIVCAAVWVLVLSGSLPTGFMLNSTNKLENNSISCF-----
GPR55   LLAIQYPLLVNHFRSPRKTFGICCIIWMLVWVGSIPIYTFHRGVEG-----YKCF-HNM-
                              TM5
LPA6    -EWKSHLSKVVIFIETVGFLIPLMLNVVCSAMVLQTLRRPNTV---------LNKKKILR
GPR55   -SDSTWSARVFFPLEIFGFLLPMGIMGFCSYRSIHILLSIQGD-DQWV----RKRACIIW
                     TM6                                      TM7
LPA6    MIIVHLFIFCFCFIPYNVNLVFYSLVRTNTLKGCAAESVVRTIYPIALCIAVSNCCFDPI
GPR55   TIATNLVVFVVSFLPVHLGLFLQFLVRNGFILNCRVKQGISLFLQLSLCFSNINCCLDVF

LPA6    VYYFTSETIQNSASSEDLYFQ*
GPR55   CYYFAIKEFRMGIKVHRPSQV*
```

Supplementary Figure 2. Sequence alignment to generate de GPR55 receptor. Sequence alignment of the template (LPA6 receptor; 5XSZ.pdb) used to generate the tertiary structure of the GPR55 receptor. The seven transmembrane helices in the structure of the LPA6 receptor are indicated by cyan rectangles. the most conserved residues in each TM is colored blue.

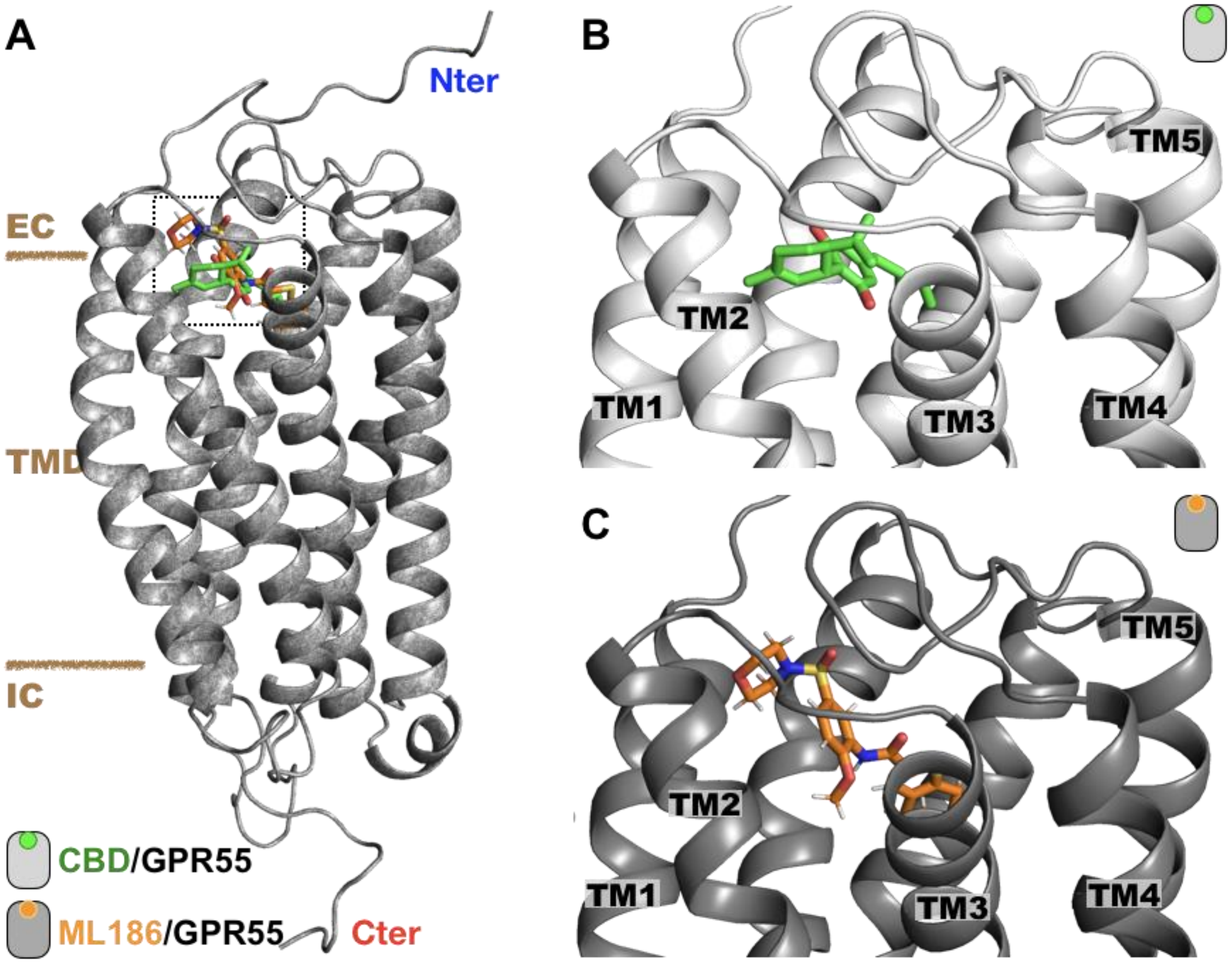

Supplementary Figure 3. Initial complex structures of the CBD/GPR55 and ML186/GPR55 complexes. (A) Lateral view of the CBD/GPR55 and ML186/GPR55 complexes. Ligand binding site of (B) the CBD compound and (C) the ML186 agonist selected from the docking calculations.

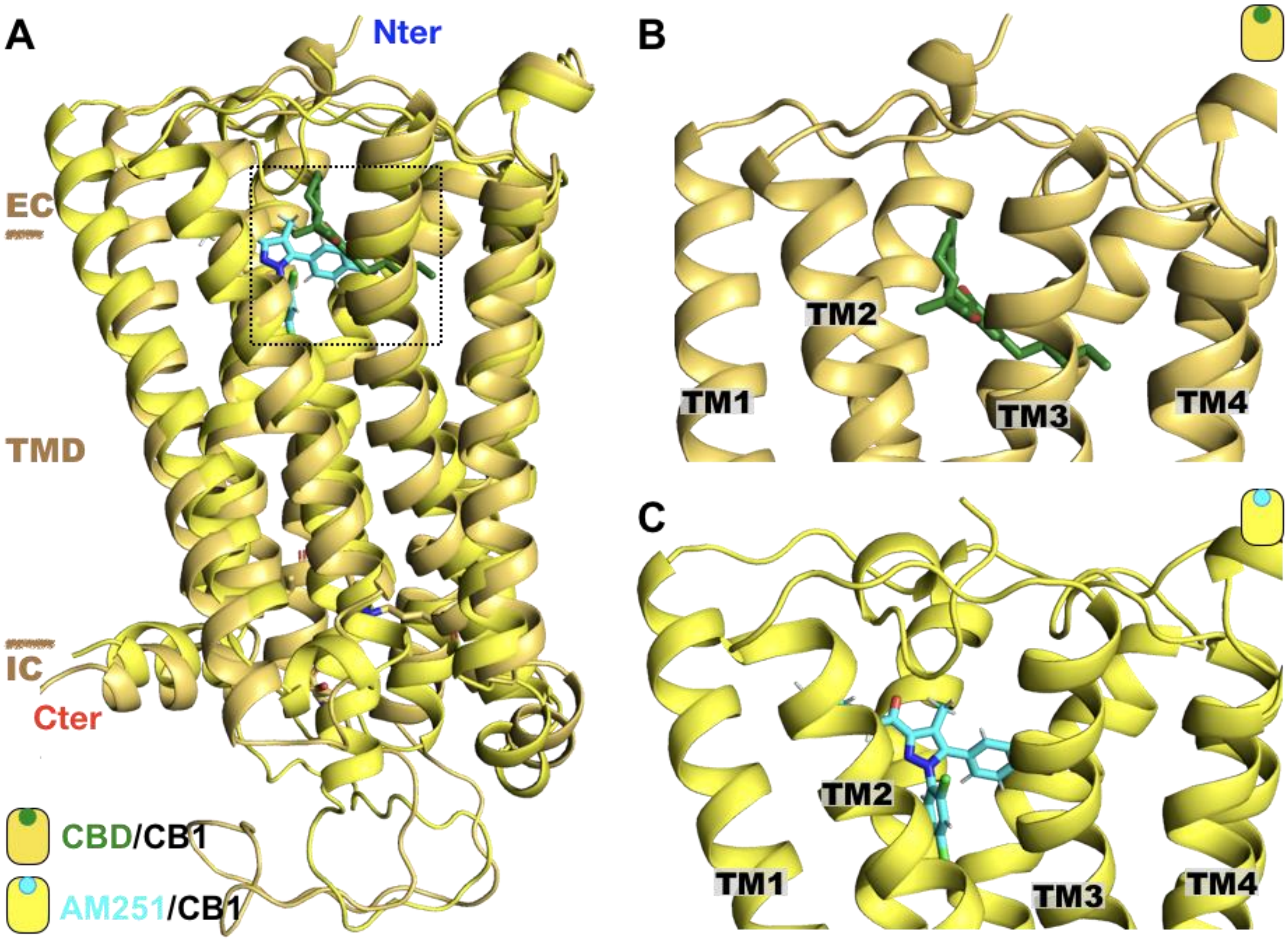

Supplementary Figure 4. Initial complex structures of the CBD/CB1 and ML186/CB1 complexes. (A) Lateral view of the CBD/CB1 and AM251/CB1 complexes. Ligand binding site of (B) the CBD compound and (C) the AM251 antagonist selected from the docking calculations.

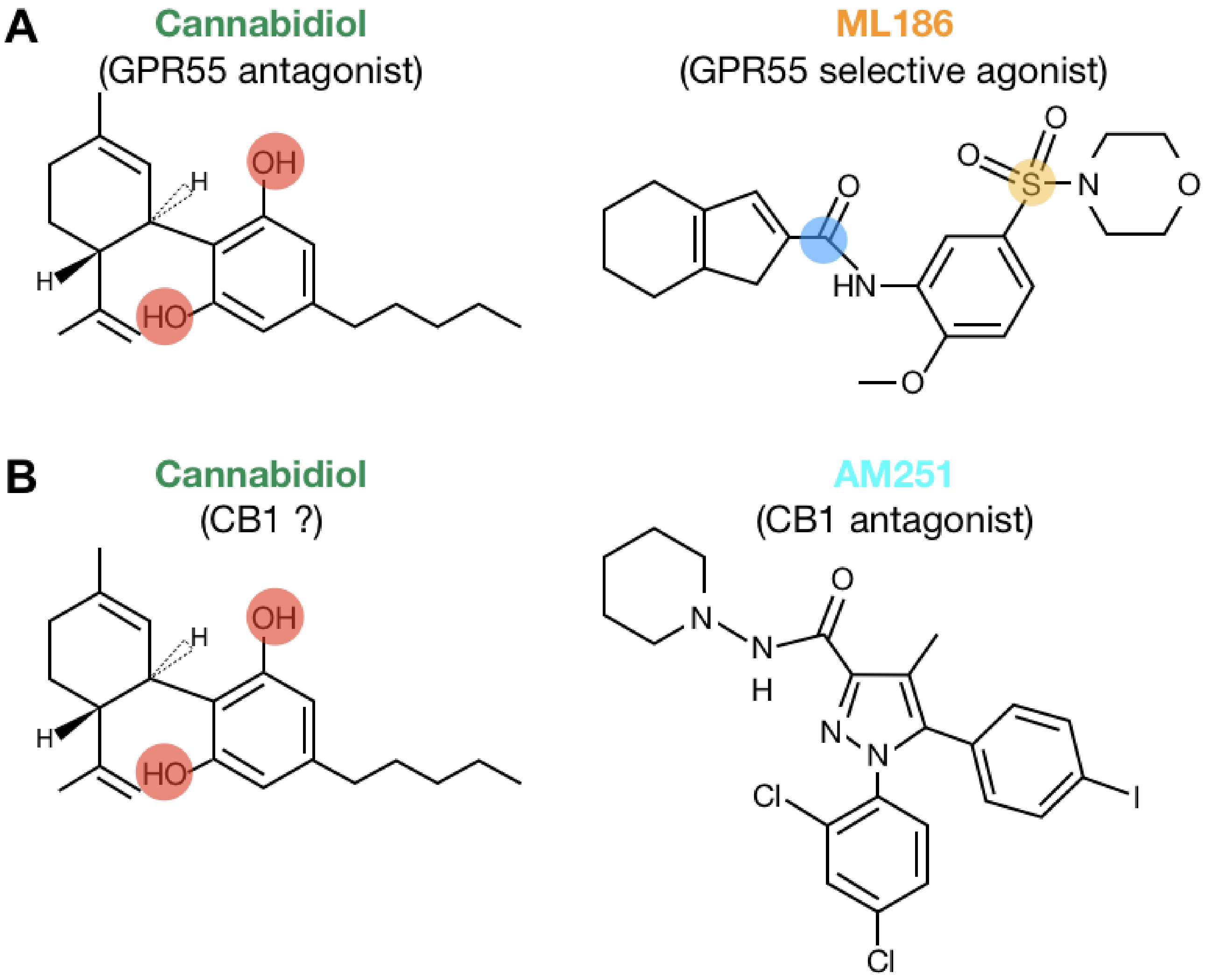

Supplementary Figure 5. Ligands investigated in this work. (A) The two ligands that modulate the function of the GPR55 receptor. CBD is an antagonist while the ML186 ligands acts as a selective GPR55 agonist. (B) The two ligands that were complex with the CB1 receptor. AM251 is an antagonist of the CB1 receptor while the function of the CBD on the CB1 remains unclear. The hydroxyl groups of CBD are indicated by a red circle. The amide carbon in the ML186 ligands is highlighted by a blue circle. The sulfur group from the thiophene ring is colored yellow.

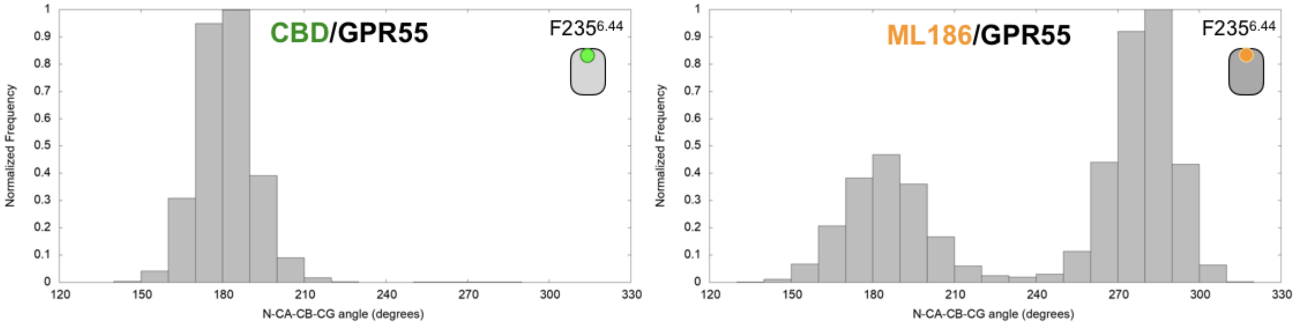

Supplementary Figure 6. Distribution of the rotameric states of F235$^{6.44}$ in both GPR55 complexes. Distributions of the N-CA-CB-CD dihedral angle of the F235$^{6.44}$ residue in the CBD/GPR55 and ML186/GPR55 complexes.
